# Multi-Response Extraction Optimization Based on Anti-Oxidative Activity and Quality Evaluation by Main Indicator Ingredients Coupled with Chemometric Analysis on *Thymus quinquecostatus* Celak

**DOI:** 10.3390/molecules23040957

**Published:** 2018-04-19

**Authors:** Yan-Li Chang, Meng Shen, Xue-Yang Ren, Ting He, Le Wang, Shu-Sheng Fan, Xiu-Huan Wang, Xiao Li, Xiao-Ping Wang, Xiao-Yi Chen, Hong Sui, Gai-Mei She

**Affiliations:** 1 School of Chinese Pharmacy, Beijing University of Chinese Medicine, Beijing 100102, China; 20160931845@bucm.edu.cn (Y.-L.C.); shenmeng44@163.com (M.S.); renxueyang1996@163.com (X.-Y.R.); heting0572@126.com (T.H.); wangle17@126.com (L.W.); fanshusheng1993@163.com (S.-S.F.); wangxiuhuan12340@163.com (X.-H.W.); lixiaocherish@sina.com (X.L.); wangxiaopingcx@163.com (X.-P.W.); chenxiaofly1209@126.com (X.-Y.C.); 2 School of Chinese Pharmacy, Ningxia Medical University, Yinchuan 750004, China

**Keywords:** *Thymus quinquecostatus* Celak, antioxidative activity, response surface analysis, thyme, quantitative analysis, UPLC-LTQ-Orbitrap MS^n^, HPLC

## Abstract

*Thymus quinquecostatus* Celak is a species of thyme in China and it used as condiment and herbal medicine for a long time. To set up the quality evaluation of *T. quinquecostatus*, the response surface methodology (RSM) based on its 2,2-Diphenyl-1-picrylhydrazyl (DPPH) radical scavenging activity was introduced to optimize the extraction condition, and the main indicator components were found through an UPLC-LTQ-Orbitrap MS^n^ method. The ethanol concentration, solid-liquid ratio, and extraction time on optimum conditions were 42.32%, 1:17.51, and 1.8 h, respectively. 35 components having 12 phenolic acids and 23 flavonoids were unambiguously or tentatively identified both positive and negative modes to employ for the comprehensive analysis in the optimum anti-oxidative part. A simple, reliable, and sensitive HPLC method was performed for the multi-component quantitative analysis of *T. quinquecostatus* using six characteristic and principal phenolic acids and flavonoids as reference compounds. Furthermore, the chemometrics methods (principal components analysis (PCA) and hierarchical clustering analysis (HCA)) appraised the growing areas and harvest time of this herb closely relative to the quality-controlled. This study provided full-scale qualitative and quantitative information for the quality evaluation of *T. quinquecostatus*, which would be a valuable reference for further study and development of this herb and related laid the foundation of further study on its pharmacological efficacy.

## 1. Introduction

Cardiovascular disease (CVD) is the major cause of death and remains the No.1 killer in the world. It is known that oxidative stress is the main reason resulted in CVD. To find antioxidants from natural sources for clinic preventive medicine has become a hot issue [1,2,3,4]. Due to growing concerns among consumers about food safety, natural antioxidants have been searched to replace chemical additives [5,6]. It was reported that a great number of spice, aromatic, and medicinal plants containing abundant phenolic compounds exhibits obviously antioxidant properties [5,7,8].

Thyme as a well-known fragrant plant is a kind of spice approved by the International Standard Organization [9]. *Thymus quinquecostatus* Celak is a variety of thyme in China. It is primarily distributed in China, Korea, and Japan [10]. This plant has been used as a food additive in cooking bacon and sausage because of its natural antiseptic action and was traditionally used as a tea-like beverage to dissipate heat and expel poison in China [11,12,13,14,15,16,17]. It also has been widely used for the treatment of arthritis, kaschin-beck disease, acute gastroenteritis, and chronic stomachaches in the clinic nowadays [18].

It is rich in essential oil of *T.quinquecostatus*, which has been proved to have strong antioxidant capacity [19,20,21]. When compared with its volatile oil, *T. quinquecostatus* in-volatile part is poorly investigated, and there are few reports on its chemical composition and pharmacological activity [22,23,24]. Our group reported that *T. quinquecostatus* had a significant anti-cerebral ischemia effect. Several flavonoids and phenolic acids were isolated from its ethanol extract for the first time also displayed obvious anti-cerebral ischemia effect and applied in many fields of clinical medicine, especially scutellarin and danshensu as a most common medicament for the treatment of cerebral ischemia [23,25,26]. According to the free radical theories, there is a great possibility that the anti-cerebral ischemia effect was due to its ability of eliminating free-radicals [27,28,29].

In this study, for the scavenging free radicals activity being quick, easy to operate, and get clear phenomenon, we had attempted to develop a reliable method for the extraction of *T. quinquecostatus*, according to the scavenging free radicals activity by response surface methodology (RSM). An UPLC-MS^2^-based method was developed to detect the possible presence of 35 commercially available phenolic acids and flavonoids in this extract. Then, an effective HPLC method was established for the multi-component quantitative analysis, which possesses good antioxidant activity. Accordingly, chemometrics methods, principal components analysis (PCA) and hierarchical clustering analysis (HCA) were utilized to probe the relation between growing areas or harvest times, and the multi-component contents of this plant.

## 2. Results and Discussions

### 2.1. Optimization of Extraction Conditions

The RSM is a combination of mathematical and statistical techniques that is useful for modelling and problem analysis. The objective is to optimize this response, as influenced by several variables. In the present study, RSM was used to optimize the extraction conditions and obtain the maximal response variables of *T. quinquecostatus* for the first time. In preliminary experiments, ethanol concentration, solid-liquid ratio, and extraction time were considered to be factors influencing the plant’s free-radical scavenging ability. The three factors were inspected and the result was shown in Table 1.

The experimental data were fitted to a second-order polynomial model, which can be described as the following Equation (1):

Y = 205.01 + 11.04 X_1_ + 43.14 X_2_ + 2.11 X_3_ − 38.98 X_1_X_2_ − 8.07 X_1_X_3_ + 64.72 X_2_X_3_ − 24.24 X_1_^2^ + 122.96 X_2_^2^ + 35.14 X_3_^2^ (1)

where Y is the measured response variables (IC_50_ value) and X_1_, X_2_ and X_3_ are the independent variables.

For any term in the model, a large regression coefficient and a small *P*-value indicated a more significant effect on the respective response variables. The ANOVA was used to assess the significance of each factor and interaction terms (Table 2). The model was found to be highly significant, as evidenced by the results of an F-test, which gave an F-value of 1655.45 (*p* < 0.0001). The ANOVA of the regression model demonstrated that this model was highly significant (R^2^ = 0.9989, *p* < 1%). $R_{\mathrm{Adj}}^{2}$ (Adjusted R^2^) provided a measure of how much variability in the observed response values could be explained by the experimental factors and their interactions. In this study, the $R_{\mathrm{Adj}}^{2}$ (0.9949) was in reasonable agreement, showing that the regression model could be used to explain 99.49% variability in the test data. In the assay process, approximately 0.5% of the results cannot be explained by the model. Moreover, the adequate precision (133.002) showed remarkable signal (>4). The F-value (2.36) and *P*-value (0.2128) for “lack-of-fit” indicated that the “lack-of-fit” was insignificant relative to the pure error. These data also indicated that the model equation was adequate for predicting response under any combination of the variables. On the basis of Table 3, all of the variables except extraction time (X_3_, *p* > 0.05) have greatest effect on the extraction efficiency (*p* < 0.01). These results indicated that the model had a good representative and could thus be used to predict the actual experiment results.

**Figure 1:**
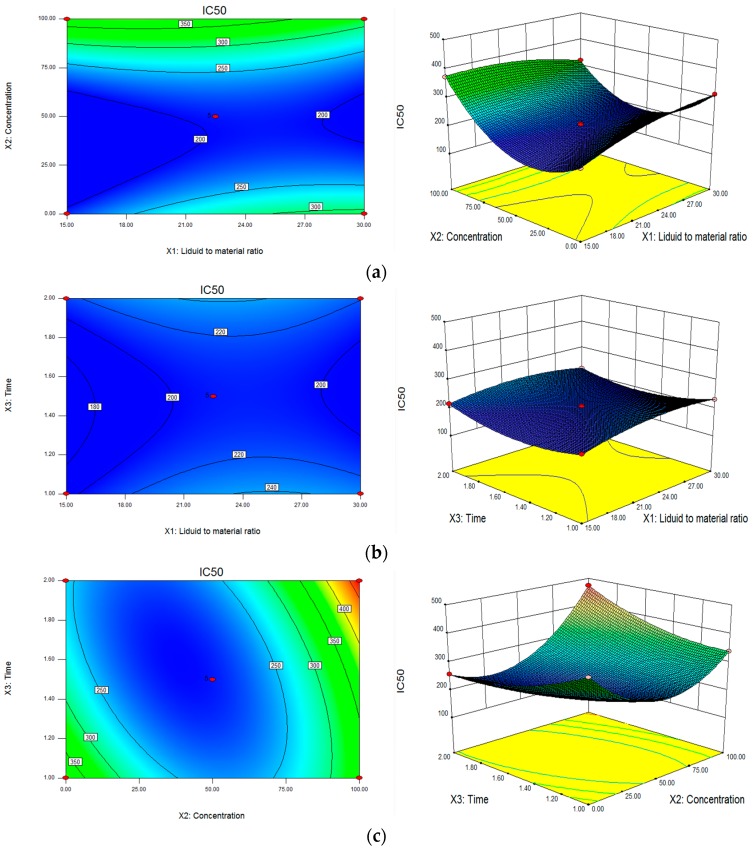
Contour plots and response surface showing the effects of the three factors on 2,2-Diphenyl-1-picrylhydrazyl (DPPH) responses of *T. quinquecostatus* extract. (**a**) ethanol concentration and liquid mass ratio; (**b**) extraction time and liquid mass ratio; and, (**c**) extraction time and ethanol concentration.

Three-dimensional plots give a comprehensive picture of the behaviors on the prediction variances throughout a region and the quality of the predicted responses that were obtained by Box-Behnken Design (BBD). Figure 1a–c, it clearly illustrated that the eliminating 2,2-Diphenyl-1-picrylhydrazyl (DPPH) capacity was mainly affected by the liquid mass ratio and ethanol concentration. The antioxidant capacity increased with the liquid mass ratio and ethanol concentration, and then decreased with further increases. The highest clearing DPPH radical was obtained at an ethanol concentration of approximately 42.32% and a liquid mass ratio of 17.51:1. In order to carry out future research on *T. quinquecostatus* and for the convenience of industrial production, the optimal ethanol concentration and liquid-solid ratio was changed to be 45% and 20:1, respectively. The extract of phenolic compounds depends largely on the polarity of solvent, and a combination of solvent and water is more effective than a solvent alone. As the ethanol concentration increased, the dielectric constant and energy that is required for breaking the water arrangement decreased, not only more phenolic compounds were extracted, but also more impurities [30]. From the Figure 1b,c, we assumed that the extraction time had nonsignificant influence on the antioxidant capacity. The trapping-DPPH action increased when the extraction time increased from 1 h to approximately 1.5 h, and then this tendency was interrupted. The result may be explained by the fact that the prolonged extraction time may cause the decomposition of active compounds, resulting in the loss of active compounds [31]. Thus, the best extraction time was 1.5 h. In conclusion, the optimum extraction process was 45% (ethanol concentration), 20:1 (liquid mass ratio), and 1.5 h (extraction time), respectively.

### 2.2. Identification of the Main Constituents of T. quinquecostatus Extract

**Figure 2:**
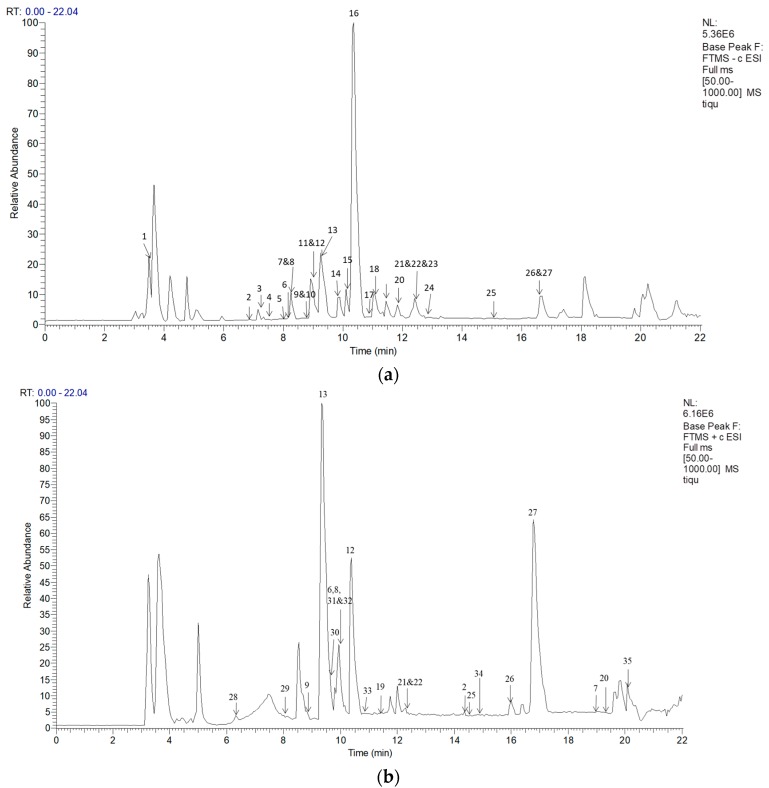
TIC chromatograms of *T. quinquecostatus* ethanol extract. (**a**) TIC chromatogramin negative ion mode; and, (**b**) TIC chromatogram in positive ion mode.

In this study, to explore the chemical compounds’ structural diversity in the extract using the optimum extraction process, an UPLC-MS^2^-based method was developed. In order to get adequate structural information of the chemical constituents in this extract and to reveal as many chemical compounds as possible, both positive and negative modes were employed for the comprehensive analysis. Finally, a total of 35 compounds were detected by Mass profiler (Figure 2) and were identified by comparing their retention time, accurate mass data, and the information of fragmentation with reported reference (Table 3 and Table 4), including 12 phenolic acids and 23 flavonoids that are characteristic of thymus species.

#### 2.2.1. Characterization of Phenolic Acids

In this work, a total number of 12 phenolic acids were identified in the extract. Peaks **3**, **5** were identified as danshensu, vanillic acid, by comparing with the reference standards. They all had the characteristic fragment ions that were generated by the neutral loss of H_2_O (18 Da) or CO_2_ (44 Da), which indicated the existence of carboxyl in molecular structures. The fragmentation behaviors of peaks **1**, **11**, **12**, and **23** were similar with that of danshensu, but the quasi-molecular ions [M − H]^−^ of them were different. According to the MS^2^ information achieved and the chemical constituents that were reported in literatures [32,33], they were identified as paeonol, hydroxybenzoic acid, caffeic acid, and ethyl caffeate, respectively. Peak **4**, which had the quasi-molecular ion at *m*/*z* 153 in negative ion mode, was tentatively characterized as gentisic acid or protocatechuic acid, due to lacking of information [33,34].

Thereinto, danshensu, and caffeic acid are the basic components in the extract. Due to the loss of 198 Da and 180 Da, peaks **15**, **16**, **17**, **33** were inferred to be condensed by different numbers of danshensu and caffeic acid. Meanwhile, the characteristic fragment ions of peak **6** were the ions at *m*/*z* [M − H − 180]^−^ and [M − H − 192]^−^ in MS^2^ spectrum, which were important to estimate the phenolic acid containing caffeic acid and quinic acid. Finally, according to the different quasi-molecular and when comparing with the data in the literature [35,36,37,38], peaks **6**, **15**, **16**, **17** and **34** were identified as chlorogenic acid, salvianolic acid A, rosmarinic acid, salvianolic acid C, and methyl rosmarinate, respectively.

#### 2.2.2. Characterization of Flavonoids

**Figure 3:**
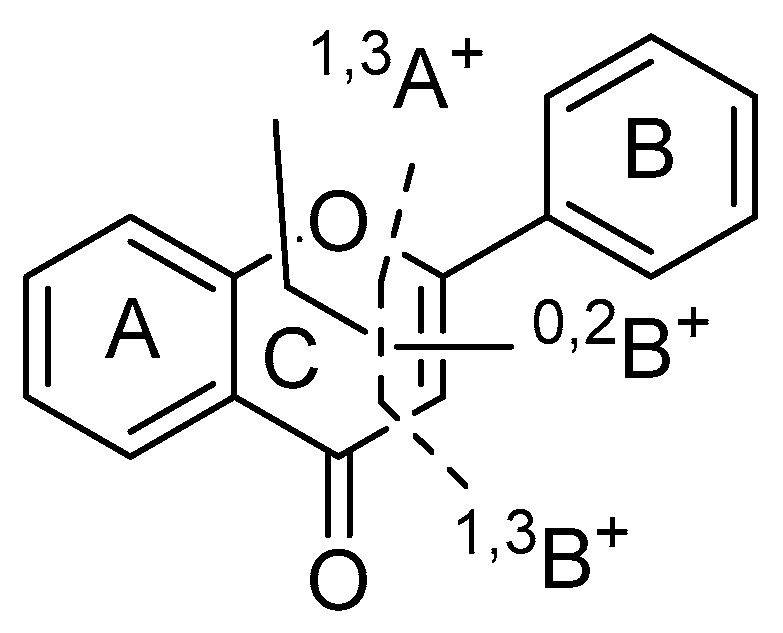
Cleavage at C ring of flavone.

Under the conditions of soft-ionization, the mass fragmentation of parent nucleus of flavonoids occurs on the C ring in general, producing the corresponding fragment ions of ^1,3^A^+^, ^1,3^B^+^, ^0,2^B^+^ (Figure 3) [39]. In this study, most of flavones had the characteristic fragment ions through the retro Diels-Alder (RDA) reaction. In the MS^2^ spectrum of peak **23** in negative ion mode, a product ion at *m*/*z* 121 [^0,2^B^+^ + OH]^−^ was generated. Finally, when comparing with the reference standards, peak 23 was identified as apigenin.

There were 23 flavonoids that were identified in the *T. quinquecostatus* extract, including eight *O*-glycosyl flavonoids, nine polyhydroxy flavones, and six polymethoxy flavones. The loss of 15 Da (CH_3_), 18 Da (H_2_O), 31 Da (CH_3_O), and 28 Da (CO) is characteristic of poly-substituted flavones. Among them, peaks **26** and **27** were characterized as xanthomicrol and cirsilineol. The identical quasi-molecular ions at *m*/*z* 345 [M + H]^+^ and similar fragmentation make it difficult to distinguish them. Yet, the flavonoids containing methoxyl at 6- and 8- will generate a strong ion [M − CH_3_]^+^, and then product ion [M − 43]^+^ by losing CO consecutively. According to this rule, peak **26** that had the product ion at *m*/*z* 330 as the base peak in MS^2^ spectrum by the loss of methyl was assigned as xanthomicrol, which is a potentially previously unrecognized compounds in the thyme genus. The loss of a hexoside residue (162 Da) or (176 Da) usually occurs on *O*-glucoside or *O*-glucuronide flavonoids and the aglycone [M − 162]^−^ was the base peak in MS^2^ spectrum. Based on the MS^2^ information achieved, 6 *O*-glucoside flavonoids and 2 *O*-glucuronide flavonoids were identified.

### 2.3. Optimization of HPLC Conditions

Based on the results of “2.2”, the compounds danshensu (peak **3**), vanillic acid (peak **4**), chlorogenic acid (peak **6**), galuteolin (peak **10**), scutellarin (peak **13**), and apigenin (peak **23**) were the characteristic constituents in each sample. Pharmacological researches show that they have good abilities of free radical scavenging, activating blood, and removing stasis [40,41,42]. Therefore, a HPLC method was developed to analyse the above six compounds.

**Figure 4:**
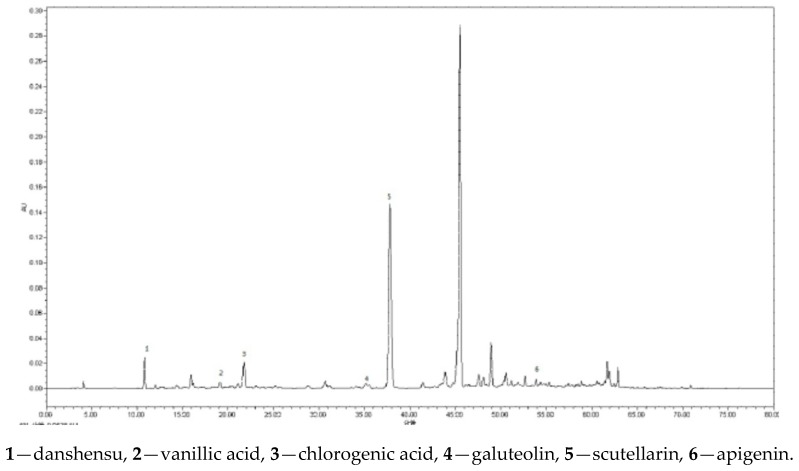
The HPLC fingerprints for alcohol extract of *T. quinquecostatus*.

*T. quinquecostatus* secondary metabolites are difficult to separate because of their complex compositions and their similar physicochemical properties. So to obtain chromatograms with good separation, the different mobile phases comprising of acetonitrile–water and methanol–water in various proportions were compared under different gradient elution modes, while they failed to produce satisfactory separation of the six analytes. Acetic acid and phosphoric acid additives were therefore investigated, and it was observed that phosphoric acid could improve the separation. The effects of flow rate and detection wavelength were also investigated, and the results showed that the separation was optimal at 1.0 mL/min and 294 nm, respectively. Finally, a gradient elution program was selected to ensure that each run was completed within 75 min. In addition, 25 °C was chosen as the column temperature. After many tests, excellent separations of the six compounds were achieved in a single run. The representative chromatogram is shown in Figure 4.

### 2.4. Validation of HPLC

#### 2.4.1. Linearity

The parameters of the linear calibration curve with the R^2^, linear range, and regression equation of the six compounds are listed in Table 5. Consequently, each coefficient of determination (R^2^) was greater than or equal to 0.9990, as determined by least squares analysis, suggesting a good linearity between the peak areas (y) and the compound concentrations (x) over a wide concentration range.

#### 2.4.2. Precision, Repeatability, Stability and Accuracy

The intra- and inter-day precisions of the 6 active components ranged from 1.28% to 1.82% and 0.21% to 1.96%, respectively, indicating that the method described was sufficiently precise enough for the quantitative evaluation of the analytes in thyme. Repeatability (RSD < 2%) demonstrated that the developed analytical method was reproducible for all of the components that were examined. The sample solutions were stable within 48 h (RSD < 2%). Average recoveries of the investigated targets ranged from 96.72% to 101.67%, and the RSD was less than 2% (*n* = 9), which demonstrated that the developed HPLC method was sufficiently reliable and accurate for the measurement of the compounds analyzed. The results of precision, repeatability, stability, and accuracy are shown in Table 5.

### 2.5. Quantitative Determination of 6 Compounds

The developed HPLC method was successfully applied to simultaneously determine the six major active components in the samples of *T. quinquecostatus* from different habitats and harvest times. Remarkable differences occurred amongst the contents of the chemical markers that were analyzed in the different samples (Table 6 showed). The habitat of thyme had a great influence on the chemical constituents’ contents. Generally, the contents in *qingyang* and *yanchi* samples (both from Gansu province) were higher than that of the *jingbian* samples (Shaanxi province). Notably, the highest contents of danshensu and scutellarin could reach to 11.080 and 4.472 mg/g, respectively. danshensu and scutellarin were the most abundant bioactive components of the six components, having a strong effect on cardio-cerebrovascular diseases [43,44]. In addition, galuteolin and apigenin were scarcely detectable in some samples, suggesting that there were obvious variations amongst the test samples in chemical compositions.

### 2.6. Chemometric Analysis

Chemometrics is an analysis method for extracting valuable information from large amounts of multivariate data. The PCA and HCA were performed to further explore the relation amongst the contents of ingredients, harvest times, and habitats.

#### 2.6.1. PCA

**Figure 5:**
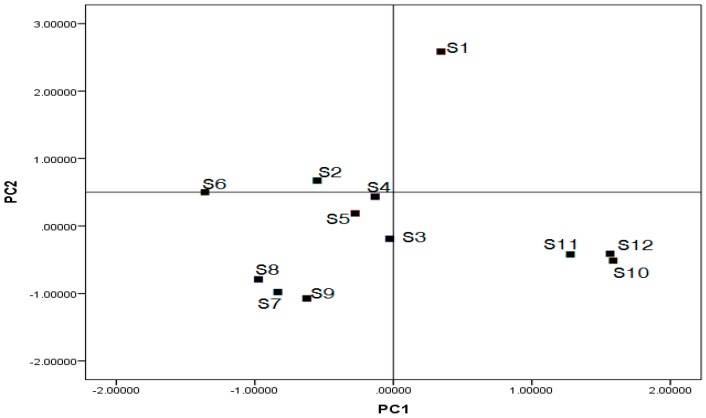
PC1–PC2 scores plot for the 12 tested samples of *T. quinquecostatus* by principal components analysis (PCA).

PCA, which is a data transformation technique, could systematically convert a large number of original variables to a set of more coherent linear combinations (called principal components, PCs) [45]. PCA is a powerful tool to reduce the dimensions of multivariate data sets [46]. In the present study, the areas of six common peaks were treated as variables. Figure 5 indicated that the first two PCs accumulated approximately 84.6% of the original data variability. The score plot indicates that the 12 samples could be classified into three groups. The S2–S5 from *qingyang* was primarily distributed in the origin of coordinates, unlike the S1, because its multi-component contents were all higher than the contents from other harvest times, particularly danshensu. This result suggested that if danshensu is the target composition, medicinal herbs harvested around July 1 in *qingyang* should be considered first. The S10–S12 from *yanchi* was primarily distributed in quadrant II, and the S7–S9 from *jingbian* was primarily distributed in quadrant Ш, except for the S6. The content of S6 was also higher than the content from other harvest times in the same habitat.

#### 2.6.2. HCA

**Figure 6:**
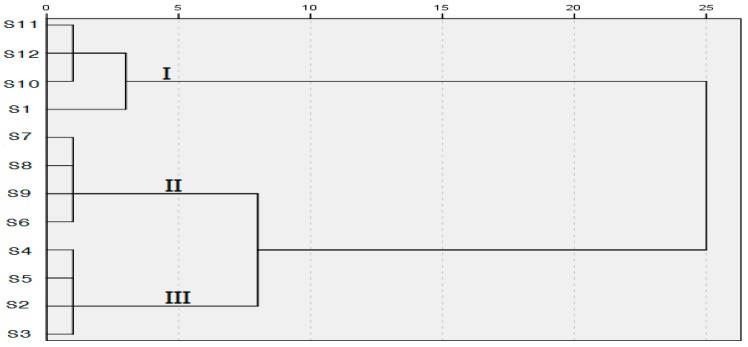
Dendrogram of hierarchical clustering analysis (HCA) for the 12 tested samples of *T. quinquecostatus*.

HCA, which is a multivariate analysis technique, is used to sort samples into groups. The between-groups linkage method as the amalgamation rule and the squared Euclidean distance, as the metric was applied to establish clusters. Figure 6 showed the resulting dendrogram, which is divided into two primary clusters. The result indicated that S1 collected from *qingyang* may be classified into group I with S10–S12 from *yanchi*, which means that S1 is more similar to *yanchi* than to other samples from *qingyang*. These results may be confirmed by the danshensu and the scutellarin contents in S1, which is more similar to S10–S12 than to other samples. As shown in Figure 6, the distance from groups II and Ш to I is longer than from group II to group Ш, which indicates that the samples in group II and group Ш were more similar. Figure 6, with a few exceptions, indicated that the contents of samples in the same habitat are quite similar to one another. The contents were quite consistent with the contents of PCA. It is also necessary to study the accumulation of the active ingredients in thyme to further optimize the best harvest season.

## 3. Materials and Methods

### 3.1. Materials and Reagents

The DPPH (2,2-Diphenyl-1-picrylhydrazyl, Sigma Co., Ltd., Croydon, UK) radical scavenging activity was determined using an ELISA microplate reader (Beijing Perlong New Technology Co., Ltd., Beijing, China) with 96-well plates (Nantong lineng Experiment Equipment Co., Ltd., Nantong, China). The *T. quinquecostatus* plant was obtained from yulin in Shaanxi province and qingyang and yanchi in the Gansu province in China. The samples were identified by Dr Shengjun Ma, department of Chinese Medicinal Resources, Beijing University of Chinese Medicine. Chlorogenic acid (Batch No. 110753), vanillic acid (Batch No. 110776-200402), scutellarin (Batch No. 110842-201207), galuteolin (Batch No. 111720-201307), apigenin (Batch No. 111901-201102), and danshensu (Batch No. 110855-201311) were reference compounds. Acetonitrile of HPLC-grade and LC-MS-grade was purchased from Fisher Scientific (Fair Lawn, NJ, USA). Phosphoric acid (Tianjin Fuchen Chemical Reagents Factory, Tianjin, China) and formic acid (Beijing Chemical works, Beijing, China) were HPLC grade. Ultra-pure waterused throughout the experiment was obtained from Guangzhou Watsons Food & Beverage Co., Ltd. (Guangzhou, China).

### 3.2. Optimization of Extraction Conditions

#### 3.2.1. Design of the Extraction Method

RSM was employed to optimize the extraction conditions in terms of antioxidant activity. The design using a three-factor, three-level BBD comprised of 17 experimental points. The three independent variables examined were solid-liquid ratio (X_1_), ethanol concentration (X_2_), and extraction time (X_3_). The actual values of each variable, which were predetermined by preliminary experiments, were coded at three levels (−1, 0, 1) for statistical analysis (Table 7).

#### 3.2.2. DPPH Radical Scavenging Activity Assay

The DPPH radical scavenging activity was determined, according to the method that was reported by Sharififar and others with some modifications [47]. An equal amount of (1 mL of 0.15 mg) DPPH solution in 70% ethanol was mixed with 0.1 mL *T. quinquecostatus* extract in 70% ethanol in various concentrations. After being mixed, the solution was allowed to reach a steady state at room temperature for approximately 1.5 h. The DPPH radical scavenging activity was determined by the absorbance at λ = 517 nm using a microplate reader and was calculated by the following Equation (2):

DPPH radical scavenging activity = (A_0_ − A_1_)/A_0_ × 100% (2)

where A_0_ is the absorbance of the control (blank, without extract) and A_1_ is the absorbance of the mixture with the extract.

### 3.3. Preparation of Sample Solution

Sample preparation was conducted according to the above optimum extraction process with an ultrasonic cleansing bath. In brief, the crushed sample (approximately 2.0 g), accurately weighed, was transferred into a 100 mL conical flask with a stopper, and 40 mL of 45% ethanol solution was added, and was then ultrasonically extracted at room temperature for 1.5 h. The extracted solution was concentrated and filtered through 0.22 µm membrane filters before analysis. The contents of the selected compounds in *T. quinquecostatus* were obtained from the corresponding calibration curves.

### 3.4. Preparation of Standard Solution

Standard stock solutions of the six reference standards, vanillic acid (12.9 µg/mL), chlorogenic acid (100.0 µg/mL), danshensu (150.0 µg/mL), galuteolin (20.0 µg/mL), scutellarin (200.0 µg/mL), and apigenin (20.0 µg/mL) were prepared by dissolving the respective working standard substance in alcohol. The solutions were then diluted with alcohol to the concentrations required. All of the solutions were stored at 4 °C before use.

### 3.5. Instrument and UPLC-MS/MS Conditions

UPLC separations were optimized and finally performed on a Themo DIONEX UltiMate 3000 system (Thermo Fisher Scientific, Waltham, MA, USA) using a reverse-phase analytical column (3.6 μm, 4.6 × 150 mm, XDB-C_18_, Agilent, Santa Clara, CA, USA) at 30 °C with a 1.0 mL/min flow rate. 1 μL test solution was injected into the UPLC system. The mobile phase comprised acetonitrile (A) and 0.1% formic acid (B). The linear eluting gradient was as follows: 0–5 min, 5–30% A; 5–8 min, 30–52% A; 8–12 min, 52%A; 12–18 min, 52–95% A; 18–22 min, 95% A; 22–23 min, 95–5% A; and, 23–30 min, 5% A.

For the ESI-MS/MS experiment, a Thermo LTQ-Orbitrap Velos Pro Hybrid mass spectrometer (Thermo Fisher Scientific, Bremen, Germany), equipped with an ESI source operating in auto-MS^n^ mode to obtain fragmentation in negative mode. Full scan data acquisition was performed from *m*/*z* 50 to 1000 in both negative and positive ion mode. The optimized ESI parameters in negative ion mode were set, as follows: capillary temperature of 350 °C; sheath gas flow rate of 40 arb; auxiliary gas flow rate of 10 arb; electrospray voltage of −3.5 V; and, tube lens voltage of −120 V. The electrospray voltage was 3.4 V and tube lens voltage was 120 V in positive ion mode; and other parameters were same as those of negative ion mode. The scan cycle employed a full-scan event at a resolution of 30,000. The most intense ions detected in the full-scan spectrum were selected for the data-independent scan. The collision energy for collision-induced dissociation (CID) was adjusted to 35% of the maximum. All of the instruments were controlled by the Xcalibur data system, and the data acquisition was carried out by analyst software Xcalibur 2.1 (Thermo Fisher Scientific, Waltham, MA, USA).

### 3.6. Instrument and HPLC Conditions

Quantitative analysis of 6 selected compounds were conducted on a Waters 2489 HPLC system (Waters Corporation (Shanghai), Shanghai, China), equipped with a breeze 2 data workstation, a UV detector and a manual injector. The separation was performed on a Thermo Hypersil GOLD C_18_ (5 μm, 4.6 × 250 mm, Thermo Fisher Scientific, Waltham, MA, USA) at 25 °C. The mobile phase comprising acetonitrile (solvent A) and 0.1% aqueous phosphoric acid (solvent B) was used to elute the target components with a gradient programme (0–5 min, 0–7% A; 5–l0 min, 7–13% A; 10–15 min, 13–15% A; 15–20 min, 15–18% A; 20–25 min, 18% A; 25–30 min, 18–20% A; 30–35 min, 20% A; 35–45 min, 20–28% A; 45–55 min, 28–40% A; 55–65 min, 40–80% A; 65–70 min, 80–100% A; and, 70–75 min, 100% A). The sample injection volume, flow rate, and detection wavelength were set at 10 μL, 1.0 mL/min, and 294 nm, respectively.

### 3.7. HPLC Method Validation

#### 3.7.1. Linearity

The stock solutions containing the six analytes were prepared and diluted with alcohol to the appropriate concentration for construction of calibration curves. At least six concentrations of each compound were analysed under optimum HPLC conditions in triplicate, and the calibration curves were then constructed by plotting the peak areas compared with the concentration of each analyte. The regression equations were calculated in the form of y = ax + b, where y and x correspond to the peak area and concentration, respectively. The results are presented in Table 4.

#### 3.7.2. Precision, Repeatability, and Stability

The measurement of intra- and inter-day variability was utilized to evaluate the precision of the developed method. The intra-day precision was investigated for the mixed standards using six replicates within one day, and inter-day precision was determined in triplicate for three consecutive days. Variations were expressed by RSD.

The repeatability of the established method was examined at one level, and the *T. quinquecostatus* samples were extracted and analysed in triplicate using the previously mentioned method. The repeatability was presented as RSD (*n* = 6).

The stability of the sample solution was also assessed at room temperature. The same sample solution was analysed in triplicate at 0, 2, 4, 8, 12, 24, and 48 h. RSD was considered as a measure of stability.

Results regarding the stability as well as the precision and repeatability were summarized (Table 4).

#### 3.7.3. Accuracy

A recovery test was used to evaluate the accuracy of the developed method. Three quantities (low, medium, and high) of the six authentic standards were added to a certain amount of the sample with known contents of target analytes. The mixtures were then extracted and analysed, as described above.

### 3.8. Identification and Determination of 6 Components

According to the HPLC chromatogram ofsample solution and standard solution, the peak position of each component in the *T. quinquecostatus* was determined (Figure 3). *T. quinquecostatus* plants, distributed in *qingyang*, *jingbian* and *yanchi*, were harvested. The plants were gathered from July to October to obtain 12 batch samples and they were dried in a shady area. The extract was prepared according to Section 3.3 and then analysed, as described above.

### 3.9. Data Analysis

Optimization of extraction conditions was conducted using the Design Expert software (Version 8.0.6.1, Stat-Ease Inc., Minneapolis, MN, USA) for regression analysis and variance (ANOVA) analysis. All 12 samples of *T. quinquecostatus* were classified by PCA and HCA using SPSS 20.0 software (IBM Company, Chicago, IL, USA).

## 4. Conclusions

To our best knowledge, the multi-responses optimization by RSM based on the DPPH radical scavenging activity was successfully implemented to evaluate the extraction of *T. quinquecostatus* for the first time. The established model exhibited favorable prediction ability and the alcoholic extract has a good antioxidant activity. In the extract, phenolic acids and flavonoids were main indicator ingredients according to the result of UPLC-MS/MS. Among them, xanthomicrol is firstly characterized in this herb. Then, six major active compounds were selected to analyze 12 batch samples. They were properly sorted on the basis of the active compounds’ content by PCA and HCA, and significant differences in the twelve different samples that were collected from different regions and harvest times were found. In summary, our work provides an easy approach that could markedly promote the study of the antioxidant parts of *T. quinquecostatus*, and establishes an effective quality evaluation method. Moreover, it has also the emphasized the chemical difference between samples from different growth environments, therefore contributing to the prominence of quality assurance and the efficacy of the medicinal plant.

## Figures and Tables

**Table 1:** The factors and response values of response surface method.

No.	X_1_/mL·g^−1^	X_2_/%	X_3_/h	Y (IC_50_ (μg/mL))
1	1	0	1	218.55
2	0	0	0	205.04
3	0	0	0	205.09
4	0	0	0	208.40
5	0	1	−1	338.87
6	0	−1	−1	382.21
7	0	−1	1	257.91
8	−1	−1	0	208.50
9	0	0	0	202.47
10	1	−1	0	312.52
11	0	0	0	204.07
12	1	1	0	321.01
13	−1	0	−1	197.13
14	0	1	1	473.46
15	−1	1	0	372.92
16	−1	0	1	216.58
17	1	0	−1	231.38

**Table 2:** The ANOVO analysis of DPPH free radical scavenging rate by response surface method.

Source	Quadratic Sum	Degree of Freedom	Mean Sum of Square	F Value	*p* Value
model	111,000	9	12,330.94	1655.45	<0.0001
X_1_	975.27	1	975.27	130.93	<0.0001
X_2_	14,888.48	1	14,888.48	1998.80	<0.0001
X_3_	35.74	1	35.74	4.80	0.0646
X_1_X_2_	6078.54	1	6078.54	816.05	<0.0001
X_1_X_3_	260.50	1	260.50	34.97	0.0006
X_2_X_3_	16,756.01	1	16,756.01	2249.52	<0.0001
X_1_^2^	2473.91	1	2473.91	332.13	<0.0001
X_2_^2^	63,662.73	1	63,662.73	8546.83	<0.0001
X_3_^2^	5197.91	1	5197.91	697.83	<0.0001
residual	52.14	7	9.94	-	-
lack of fit	33.31	3	11.10	2.36	0.2128
pure error	18.83	4	9.07	-	-
Total	111,000	16	-	-	-

“-” not detected.

**Table 3:** Summary of chemical constituents identified in *T. quinquecostatus* alcoholic extract responding to Figure 3a.

Peak NO.	Retention Time (min)	Formula Empirical	MW	Precurser Ions [M − H]^−^	Fragmentation	Tentative Structural Elucidation
1	3.49	C_9_H_10_O_3_	166.17	165.0395	165.0395, 147.0298, 129.0191	Paeonol
2	6.88	C_16_H_12_O_7_	316.26	315.0700	297.0604, 246.9443, 153.0193, 135.0450	Isorhamnetin
3	7.15	C_9_H_10_O_5_	198.17	197.0441	179.0347, 153.0552, 135.0447	Danshensu
4	7.33	C_8_H_8_O_4_	168.15	167.0338	149.0239, 123.0449	Vanillic acid
5	7.87	C_7_H_5_O_4_	154.12	153.0182	135.0447, 109.0293, 91.07836	Gentisic acid or Protocatechuic acid
6	8.00	C_16_H_18_O_9_	354.31	353.0844	191.0561, 173.0456, 161.0241, 135.0450	Chlorogenic acid
7	8.26	C_20_H_20_O_8_	388.37	387.1634	225.1128, 207.1027, 179.0557, 163.1128	Desmethylnobiletin
8	8.42	C_21_H_20_O_10_	432.38	431.1884	385.1869, 341.1600, 311.0554, 279.0715, 223.1341	Apigenin-7-*O*-glucoside
9	8.61	C_22_H_21_O_10_	478.40	477.0628	459.0562, 415.0665, 397.0566, 373.0568, 343.0461, 301.0355	Isorhamnetin-3-*O*-glucoside
10	8.67	C_21_H_20_O_11_	448.38	447.0887	357.0616, 327.0513, 285.0406	Galuteolin
11	8.92	C_7_H_5_O_3_	138.12	137.0235	93.0343	Hydroxybenzoic acid
12	9.02	C_9_H_8_O_4_	180.16	179.0336	135.0450	Caffeic acid
13	9.26	C_21_H_18_O_12_	462.36	461.0702	327.0508, 285.0405	Scutellarin
14	9.84	C_21_H_18_O_11_	446.36	445.0749	269.0454, 240.9279, 175.0248	Apigenin-7-*O*-glucuronide
15	10.21	C_26_H_22_O_10_	494.45	493.1100	449.1244, 383.0775, 359.0776, 313.0723, 295.0620	Salvianolic acid A
16	10.35	C_18_H_16_O_8_	360.31	359.0750	341.0663, 315.0870, 197.0458, 179.0353, 161.0247,	Rosmarinic acid
17	10.61	C_26_H_20_O_10_	492.43	491.0946	311.0568, 295.0614, 267.0664, 223.0249, 179.0351	Salvianolic acid C
18	11.07	C_15_H_12_O_6_	288.25	287.0540	269.0448, 259.0608, 243.0660, 151.0033	Eridioctyol
19	11.45	C_15_H_10_O_6_	286.24	285.0386	257.0459, 241.0497, 133.0269	Luteolin
20	11.84	C_18_H_16_O_6_	328.32	327.2159	309.2064, 291.1958, 165.0920	Salvigenin
21	12.30	C_17_H_14_O_7_	330	329.0644	314.0432, 229.1438, 211.1333	Thymusin
22	12.40	C_11_H_12_O_4_	208.21	207.0651	179.0346, 161.0240, 135.0449	Ethyl caffeate
23	12.57	C_15_H_10_O_5_	270.24	269.0450	225.0551, 149.0241, 121.0293	Apigenin
24	12.71	C_16_H_12_O_6_	300.26	299.0540	284.0326, 271.0421, 137.0270	Sorbifolin
25	14.97	C_17_H_14_O_6_	314.289	313.0696	298.0483, 283.0241, 245.0448	Ladanein or Cirsimaritin
26 or 27	16.62	C_18_H_16_O_7_	344.32	343.0803	328.0591, 313.0349	Cirsilineol or Xanthomicrol

**Table 4:** Summary of chemical constituents identified in *T. quinquecostatus* alcoholic extract responding to Figure 3b.

Peak NO.	Retention Time (min)	Molecular Formula	MW	Precurser Ions [M + H]^+^	Fragmentation	Tentative Structural Elucidation
28	6.31	C_15_H_12_O_5_	272.25	272.9965	254.9872, 245.0031, 229.0079, 210.9974, 198.9973, 185.0181, 118.9713	Naringenin
29	8.11	C_27_H_30_O_15_	594.52	595.1621	577.1669, 559.1462, 541.1353, 529.1353, 511.1248, 475.1426, 457.1143	Luteolin-*O*-rutinoside
9	8.81	C_22_H_22_O_12_	478.406	479.0795	317.0836, 303.0502	Isorhamnetin-*O*-glucoside
13	9.33	C_21_H_18_O_12_	462.36	463.0854	287.0551, 251.1245	Scutellarin
30	9.62	C_16_H_12_O_6_	300.26	301.0693	286.0468, 273.0385, 242.0498, 227.0236, 167.7154	Sorbifolin
6	9.80	C_16_H_18_O_9_	354.31	355.1710	337.1066, 203.0522, 193.1195	Chlorogenic acid
8	9.93	C_21_H_18_O_11_	446.36	447.0904	429.0816, 271.0603	Apigenin-7-O-glucuronide
31	10.15	C_27_H_30_O_14_	578.52	579.1078	517.1119, 471.1556, 399.0691, 381.0586, 337.0686, 319.0580	Apigenin-7-*O*-rutinoside
32	10.25	C_20_H_20_O_5_	340.375	341.0640	323.0548, 297.0756, 279.0649, 187.0387	Prenylnaringenin
12	10.38	C_9_H_8_O_4_	180.16	181.0492	163.0388	Caffeic acid
33	10.83	C_21_H_22_O_10_	434.39	435.1234	391.1365, 373.1256, 349.1255, 271.0421, 227.0523	Naringenin-7-*O*-glucoside
19	11.44	C_15_H_10_O_6_	286.24	287.0532	153.0182, 137.8446	Luteolin or Scutellarein
21	12.18	C_17_H_14_O_7_	330	331.0792	316.0573, 298.0466, 255.0646, 213.0387	Thymusin
22	12.28	C_15_H_10_O_5_	270.24	271.0584	135.0026	Apigenin
2	14.35	C_16_H_12_O_7_	316.26	317.1001	275.0909, 197.0441, 147.0437, 125.0232,	Isorhamnetin
25	14.41	C_17_H_14_O_6_	314.289	315.0845	300.0626, 282.0520, 196.0326, 175.6378, 154.7404	Ladanein or Cirsimaritin
34	14.83	C_19_H_18_O_8_	374.345	375.1054	360.0840, 345.0603,213.0390, 165.0342	Methyl rosmarinate
26	15.99	C_18_H_16_O_7_	344.319	345.0952	330.0732, 312.0626, 297.0390, 227.0547, 212.8993	Xanthomicrol
27	16.76	C_18_H_16_O_7_	344.319	345.0953	330.0733, 315.0498, 149.0233, 135.0439	Cirsilineol
7	18.98	C_20_H_20_O_8_	388.372	389.1206	374.0994, 359.0758, 341.0652, 328.0936, 227.0544	Desmethylnobiletin
20	19.35	C_18_H_16_O_6_	328.32	329.1000	314.0782, 301.1792, 287.1273, 269.1168, 163.0750, 135.0803	Salvigenin
35	20.08	C_19_H_18_O_7_	358.346	359.1108	344.0889, 329.0654, 311.0547, 227.0548, 182.2784	Gardenin B

**Table 5:** Linear regression data, precision, repeatability, stability, and accuracy of the six compounds.

Analyte	Regression Equation	R^2^	Linear Range (μg/mL)	Precision (RSD, %, *n* = 6)		Repeatability (RSD, %, *n* = 6)	Stability (RSD, %, *n* = 6)	Recovery (%, *n* = 9)	
				Intra-Day (*n* = 6)	Inter-Day (*n* = 3)			Mean	RSD
danshensu (**1**)	y = 1126x + 724.5	0.9990	4.68–150.0	1.82	1.29	1.49	1.28	99.98	0.75
vanillic acid (**2**)	y = 29710x − 1791.2	0.9998	0.40–12.9	1.28	1.96	1.84	1.95	99.72	1.49
chlorogenic acid (**3**)	y = 21533x − 76485	0.9995	6.25–100.0	1.67	1.11	1.84	1.21	98.34	0.91
galuteolin (**4**)	y = 13898x − 5771	0.9990	1.25–20.0	1.53	0.49	1.92	1.85	100.84	1.24
scutellarin (**5**)	y = 17290x − 63063	0.9999	6.25–200.0	1.61	0.21	1.97	1.25	98.94	0.67
apigenin (**6**)	y = 30618x − 5312	0.9999	0.625–20.0	1.88	1.70	1.56	1.60	101.67	1.30

R^2^: correlation coefficient; y: peak area; x: concentration (μg/mL).

**Table 6:** Contents of the six compounds in the 12 samples.

Sample			Content of Each Compound (mg/g)					
No.	Habitat	Harvest Time	Danshensu	Vanillic Acid	Chlorogenic Acid	Galuteolin	Scutellarin	Apigenin
S1	qingyang	7.1	11.800	0.072	0.450	0.152	3.650	0.034
S2		7.15	5.750	0.027	0.450	0.095	1.650	0.019
S3		8.1	4.380	0.018	0.850	0.062	3.310	0.013
S4		8.15	5.600	0.041	0.700	0.050	2.520	0.029
S5		9.1	5.550	0.036	1.050	0.052	2.050	0.037
S6	jingbian	8.1	2.650	0.015	0.330	0.073	0.630	0.036
S7		8.15	0.840	0.016	1.210	0.065	0.490	0.019
S8		9.1	1.230	0.007	0.550	0.019	0.580	0.017
S9		9.15	1.670	0.007	1.300	0.064	1.270	0.016
S10	yanchi	6	7.035	0.075	1.181	-	4.472	-
S11		7	9.021	0.056	1.034	-	3.612	-
S12		8	9.386	0.066	1.179	-	4.057	-

“-” not detected.

**Table 7:** The factors and levels of response surface method.

Factors	Level		
	−1	0	1
X_1_/mL·g^−1^	15	22.5	30
X_2_/%	0	50	100
X_3_/h	1	1.5	2
